# Correction to “Aggressive Disease and Poor Clinical Outcome in CEBPα‐Mutated Acute Myeloid Leukemia Patient”

**DOI:** 10.1002/ccr3.71672

**Published:** 2025-12-11

A. A. Alshammari, M. O. AlSabbagh, H. M. Alshehri, A. F. AlSwayyed, F. M. Alseraye, and A. A. Peer‐Zada, “Aggressive Disease and Poor Clinical Outcome in CEBPα‐Mutated Acute Myeloid Leukemia Patient,” *Clinical Case Reports* 13, no. 11 (2025): e71462, https://doi.org/10.1002/ccr3.71462.

In the published version of this article, the author name was incorrect.

The name appeared as follows: **Attatullah A. Alshammari**.

It should read as follows: **Abdullah Attullah Alshammari**.

We apologize for this error.

